# Encapsulation of Olive Leaves Extracts in Biodegradable PLA Nanoparticles for Use in Cosmetic Formulation

**DOI:** 10.3390/bioengineering4030075

**Published:** 2017-09-12

**Authors:** Maritina Kesente, Eleni Kavetsou, Marina Roussaki, Slim Blidi, Sofia Loupassaki, Sofia Chanioti, Paraskevi Siamandoura, Chrisoula Stamatogianni, Eleni Philippou, Constantine Papaspyrides, Stamatina Vouyiouka, Anastasia Detsi

**Affiliations:** 1 Laboratory of Organic Chemistry, School of Chemical Engineering, National Technical University of Athens, Zografou Campus, 15780 Athens, Greece; maritina.kesente@gmail.com (M.K.); eleni29@hotmail.com (E.K.); mroussaki@outlook.com (M.R.); 2 Department of Food Quality and Chemistry of Natural Products, Mediterranean Agronomic Institute of Chania (Centre International de Hautes Etudes Agronomiques Mediterraneennes), 73100 Chania, Crete, Greece; slim.blidi@maich.gr (S.B.); sofia@maich.gr (S.L.); 3 Laboratory of Food Chemistry and Technology, School of Chemical Engineering, National Technical University of Athens, Zografou Campus, 15780 Athens, Greece; schanioti@gmail.com (S.C.); psiamandoura@hotmail.com (P.S.); 4 Korres Natural Products, Drosini 3, 14452 Athens, Greece; chrisoula.stamatogianni@korres.com (C.S.); lena.philippou@korres.com (E.P.); 5 Laboratory of Polymer Technology, School of Chemical Engineering, National Technical University of Athens, Zografou Campus, 15780 Athens, Greece; kp@cs.ntua.gr (C.P.); mvuyiuka@central.ntua.gr (S.V.)

**Keywords:** olive leaves, encapsulation, nanoparticles, delivery, biodegradable polymers, poly(lactic acid), natural antioxidants, cosmetics

## Abstract

The aim of the current work was to encapsulate olive leaves extract in biodegradable poly(lactic acid) nanoparticles, characterize the nanoparticles and define the experimental parameters that affect the encapsulation procedure. Moreover, the loaded nanoparticles were incorporated in a cosmetic formulation and the stability of the formulation was studied for a three-month period of study. Poly(lactic acid) nanoparticles were prepared by the nanoprecipitation method. Characterization of the nanoparticles was performed using a variety of techniques: size, polydispersity index and ζ-potential were measured by Dynamic Light Scattering; morphology was studied using Scanning Electron Microscopy; thermal properties were investigated using Differential Scanning Calorimetry; whereas FT-IR spectroscopy provided a better insight on the encapsulation of the extract. Encapsulation Efficiency was determined indirectly, using UV-Vis spectroscopy. The loaded nanoparticles exhibited anionic ζ-potential, a mean particle size of 246.3 ± 5.3 nm (Pdi: 0.21 ± 0.01) and equal to 49.2%, while olive leaves extract release from the nanoparticles was found to present a burst effect at the first 2 hours. Furthermore, the stability studies of the loaded nanoparticles’ cosmetic formulation showed increased stability compared to the pure extract, in respect to viscosity, pH, organoleptic characteristics, emulsions phases and grid.

## 1. Introduction

Oxidative stress is one of the main factors which cause skin aging [1,2,3,4,5,6,7,8,9]. For its defence, skin is equipped with enzymes and endogenous antioxidants in order to control the reactive oxygen species (ROS) in balance and repair the damage caused by them. However, over time this defensive mechanism of the skin weakens [2,7]. Plant and herb extracts, rich in natural antioxidants, have been widely used in topical formulations to help skin’s endogenous protection system from oxidative damage [2,3,4,6,8,9,10,11,12].

From ancient times in the regions around the Mediterranean Sea and the islands therein, olive leaves extract (OLE) has been widely used in folk medicine [13,14,15,16]. The main components of the leaves of the olive tree (*Olea europea*) are secoiridoids like oleuropein, ligostroside and dimethyloleuropein. Furthermore, they contain flavonoids (such as luteolin-7-glucoside, apigenin, diosmetin, rutin) as well as phenolic compounds (such as tyrosol, caffeic acid, vanillin, vanillic acid, hydrotyrosol) [15,16,17].

Among this plethora of active components, oleuropein is the main constituent of olive leaves extract, and presents a very broad variety of properties; anti-inflammatory, antimicrobial, antioxidant, cardio protective, antiviral, anti-ischemic and hypolipimedic [15,16,17]. Oleuropein can be hydrolysed to hydroxytyrosol and elenolic acid or to oleuropein aglycon and glucose [15,18].

In spite of this wide spectrum of properties, the use of olive leaves extract is limited due to its sensitivity to temperature, light and oxidation, while it presents low aqueous solubility and a bitter taste [19]. A way to overcome these limitations is to encapsulate the extract into biodegradable polymeric nanoparticles (NPs) [20,21,22,23,24,25,26]. In general, encapsulation strategies are being constantly developed in order to face a significant number of drawbacks when using pure active compounds systemically or topically. The main benefits are: (i) protection of encapsulated compounds from degradation and adverse environments; (ii) improvement of active compound physicochemical characteristics; (iii) controlled release; and (iv) precision targeting, all resulting in enhanced effectiveness and bioavailability.

Especially for the cosmetics and personal care markets, the topical application of the relevant formulations requires the successful delivery of active ingredients through the skin’s lipid barrier, that is, *stratum corneum*; it is the upper 10–20 μm layer of the skin, being the first barrier in dermic diffusion with a structure similar to “plastic wrap” [20]. Transport through the *stratum corneum* is restricted to molecules of low molecular mass (<500 Da) and moderate lipophilicity (partition coefficients, log K_o/w_ values between 1 and 3), having enough solubility in the liquid domain of the *stratum corneum* while still having sufficient hydrophilic nature to allow partitioning into the epidermis [27]. In healthy skins, loaded micro/nanocarriers penetrate into the *stratum corneum* and hair follicle canals, releasing the encapsulated ingredients there, with no evidence for the translocation of the entire particles [20,22,28,29,30,31,32].

In this study, the aim was to encapsulate the olive leaves extract in polymeric NPs, characterize the nanoparticles and define the experimental parameters that affect the encapsulation procedure. Moreover, the loaded nanoparticles were incorporated in a cosmetic formulation and the stability of the formulation was studied for a three-month period study. The polymer selected was poly(lactic acid) (PLA), which is an aliphatic polyester, lipophilic, biodegradable, biocompatible, commonly used in several clinical trials and approved by European regulatory authorities as well as FDA [24,26,29,33,34]. Finally, the emulsification-solvent evaporation technique was the chosen method for the preparation of the nanoparticles.

## 2. Materials and Methods

### 2.1. Materials

Olive tree (*Olea europaea*) leaves (1 kg) were collected from pesticide-free olive trees in the “Afidnes” region, belonged to the “Megaritiki” cultivar and were dried at room temperature, in the dark, for nine days. The dried leaf material obtained was 670.4 g. The PLA used presented viscosity-average molecular weight ($\bar{M_{v}}$) of 46,000 g/mol and was obtained from solid state hydrolysis of a commercial resin (PLI005, NaturePlast, Ifs, France) at 60 °C under acidic conditions (pH 3) [35]. Poly(vinyl alcohol) (PVA) (Alfa Aesar, Ward Hill, MA, USA), which was used as emulsion stabilizer, was of average molecular weight 88,000–97,000, 87–89% hydrolysed. All the organic solvents used were of analytical grade.

### 2.2. Methods

#### 2.2.1. Preparation of Olive Leaves Extract (OLE)

**Figure 1:**
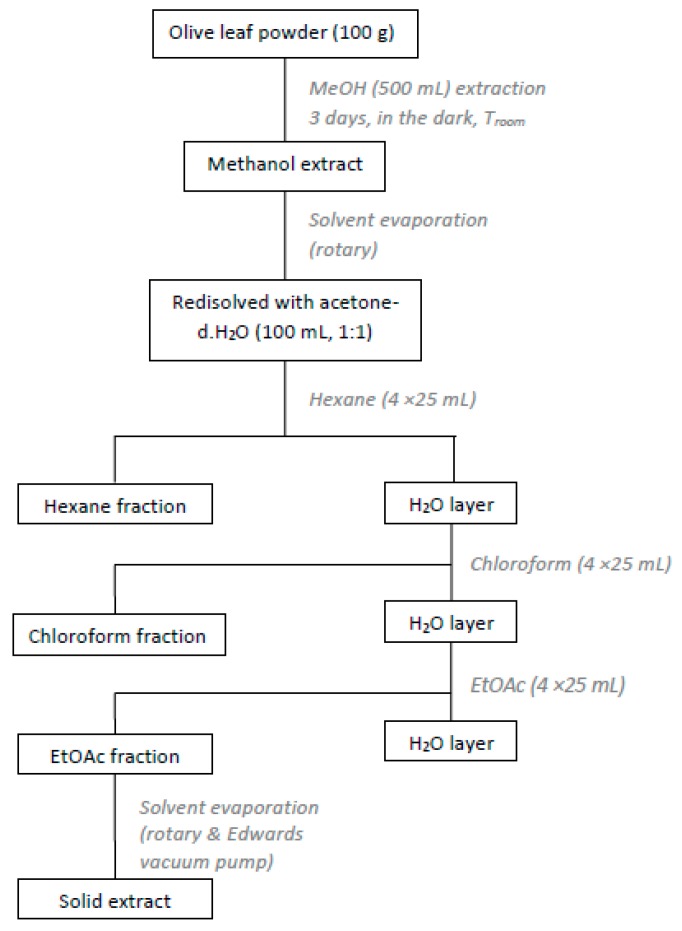
Experimental procedure for the preparation of olive leaves extract (OLE).

The dried olive leaves were extracted with organic solvents of different polarity [15]. Briefly, dried leaves (100 g) were chopped and extracted with methanol (500 mL) for 3 days, in the dark, at room temperature. Afterwards the methanol extract was filtered and concentrated under reduced pressure. The residue was re-dissolved in an acetone-water solution (100 mL, 1:1) and washed with hexane (4 × 25 mL), chloroform (4 × 25 mL) and ethyl acetate (4 × 25 mL). The ethyl acetate extracts were combined and concentrated in vacuo (Figure 1). The solid residue (2.2 g) was collected and kept at 4 °C, in dark-coloured glass vials. The procedure was repeated for the rest of the dried material. Finally, from the 670.4 g of dried olive leaves a total amount of 14.8 g of extract was obtained (yield 2.2%).

#### 2.2.2. High-Performance Liquid Chromatography Analysis of Olive Leaves Extract

HPLC-analysis was performed on a HP 1100 Series gradient HPLC system (Agilent Technologies, Santa Clara, CA, USA) equipped with Class VP chromatography data station software, a SIL-10AF autosampler, a CTO-10AS column oven (251 °C), an SPD-10AV UV Visible detector and a diode array detector (DAD) (Hewlett-Packard, Waldbronn, Germany). A column (250 × 4.6 mm) packed with 5 μm particles Hypersil C18 (MZ 156 Analysentechnik, Mainz, Germany) was used. The elution solvents consisted of aqueous 6% acetic acid and 2 mM sodium acetate (solvent A) and acetonitrile (solvent B). Gradient elution: 0–25 min, 100–50% A and 0–50% B, flow rate 0.8 mL/min; 25–26 min, 50–0% A and 50–100% B, flow rate 0.8 mL/min; 26–27 min, 0% A and 100% B, flow rate 0.8–1.2 mL/min; 27–40 min, 0% A and 100% B, flow rate 1.2 mL/min; 40–41 min, 0–100% A and 100–0% B, flow rate 1.2–0.8 mL/min, 41–45 min, 100% A and 0% B, flow rate 0.8 mL/min The injection volume was 20 μL and DAD signals were recorded at 280 nm (oleuropein) and 330 nm (vanillin and rutin) [36].

The identification of polyphenols in olive leaves extract was performed by comparison of retention times of standard solutions and confirmed with characteristic spectra using the diode array detector.

The quantification of identified phenolic compounds was carried out by external 6-point calibration pure standards. The linearity of the calibration curves was verified in each case by analysis in triplicate of standard solutions containing 100–1000 μg/mL for oleuropein, 0.08–0.8 μg/mL for vanillin and 152–1520 μg/mL for rutin. The concentration of polyphenols was expressed in mg/mL.

#### 2.2.3. Luminol Chemiluminescence Assay

A chemiluminescence method was used as described by [37]. One millilitre of borate buffer solution (50 mM, pH 9) containing CoC_l2_·6H_2_O (8.4 mg/mL) and EDTA (2.63 mg/mL) was first mixed with 0.1 mL of luminol solution (0.56 mM in borate buffer 50 mM, pH 9) and vortexed for 15 s. All samples were dissolved in DMSO. An aliquot of 0.025 mL of sample and 0.025 mL of H_2_O_2_ aqueous solution (5.4 mM) were then added into the test tube, the mixture was rapidly transferred into a glass cuvette, and the CL intensity (Io) was recorded. The instantaneous reduction in Io elicited by the addition of the sample was recorded as I and the ratio (Io/I) was calculated and plotted vs. concentration (mg/mL) of the sample. The concentration of sample (IC_50_), which is required to decrease Io intensity by 50%, was also calculated. For all measurements, a LS55 Luminescence Spectrometer-Perkin Elmer was used, keeping the lamp off and using only the photomultiplier of the apparatus. All determinations were carried out at least in triplicate and values were averaged and given along with the standard deviation (±SD).

#### 2.2.4. Preparation of PLA Nanoparticles (PLA NPs)

PLA nanoparticles were prepared by the nanoprecipitation method. For a 20% drug loading (mass of OLE per mass of polymer used, w/w) PLA (20 mg) was dissolved in acetone (2 mL), and then mixed with an OLE-MeOH solution (4 mg of OLE in 2 mL MeOH). Subsequently, the organic solution was injected in an aqueous solution of PVA (1% w/v). The emulsion was left in magnetic stirring for 10 min at 250 rpm and then in shaker at 90 rpm, overnight, for evaporation of the solvents. Afterwards, the nanoparticles formed were recovered by centrifugation. An initial centrifugation at 17,000 rpm for 20 min at 10 °C was performed. The resulting supernatant (S1) was recovered and stored in a dark-coloured container. The nanoparticle sediment was re-suspended in deionized H_2_O and centrifuged under the same conditions (17,000 rpm, 20 min and 10 °C) and the supernatant (S2) was recovered and stored. The NPs obtained were re-dispersed in doubly distilled (dd) H_2_O (1 mL) and stored at 4 °C. Unloaded (blank) NPs were prepared using the same procedure without the addition of the OLE.

#### 2.2.5. 1,1-Diphenyl-2-picryl-hydrazyl (DPPH) Radical Scavenging Assay

The DPPH method was used to determine antioxidant activity of olive leaves extract. Briefly, a solution of DPPH in methanol (0.1 M) was prepared and added (0.1 mL) to samples of different concentration OLE solution in methanol (3.9 mL, 0.03–0.4 mg/mL) and allowed to react in the dark, at room temperature. After 20 min, the absorbance values (Abs_sample_) were measured at 515 nm against a blank sample (Abs_blank_). The remaining free radical DPPH in percent was calculated using Equation (1):

(1) %RemDPPH =AbssampleAbsblank×100

In order to calculate the IC_50_ value, a graph of the remaining DPPH (% RemDPPH) against the sample concentration (C_sample_) was plotted.

#### 2.2.6. Total Phenolic Content

The total phenolic content of the OLE was determined by using the Folin-Ciocalteu method with some modifications. 100 μL of OLE or standard solution of Gallic acid (50, 100, 150, 250, 500 ppm) in MeOH were added to 500 μL of a Folin-Ciocalteu solution, followed by 1.5 mL of sodium carbonate solution. The reagents were mixed and incubated for 30 min in the dark at room temperature. Afterwards, the absorbance was measured at 765 nm in triplicate. The average data was interpolated in a gallic acid calibration curve and the total phenolic content was expressed as mg Gallic Acid Equivalents (GAE)/g of dry extract.

### 2.3. Nanoparticles (NPs) Characterization

#### 2.3.1. Determination of Particle Size, Pdi and ζ-Potential

Mean particle size, polydispersity index (Pdi) and ζ-potential of loaded and blank nanoparticles were determined via Dynamic Light Scattering (DLS) technique, using a Zetasizer Nano ZS (Malvern Instruments, Malvern, UK). The samples were prepared by dispersing 0.2 mL of nanoparticle suspension in dd. H_2_O (4 mL) resulting in off-white opaque aqueous suspensions. All measurements were performed in triplicate, at 25 ± 1 °C and results were reported as mean ± standard deviation (SD).

#### 2.3.2. Morphology

The surface morphology of the nanoparticles prepared was examined using Scanning Electron Microscopy (SEM). The analysis was performed on a NanoSEM 230 (FEI Company, Hillsboro, OR, USA) equipped with an Everhart-Thornley Detector (ETD) and a Through Lens Detector (TLD). In order to secure conductivity of the surface for clear imaging, gold sputtering was applied with a nominal thickness of 7 nm using an EMS 550X Sputter Coater (Hatfield, PA, USA).

#### 2.3.3. Encapsulation Efficiency Determination

**Figure 2:**
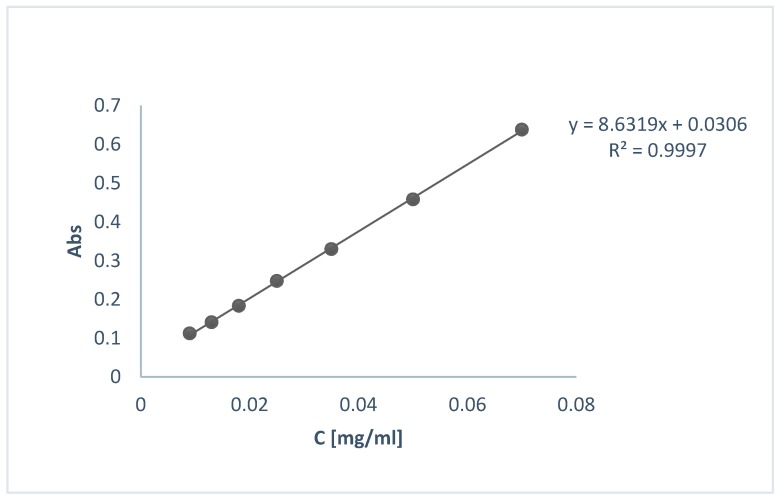
Calibration curve of OLE for the determination of EE% and in vitro release profile.

Encapsulation efficiency (EE%) was determined indirectly using UV-Vis spectroscopy. For that purpose, the supernatants (S1, S2) from the centrifugation step of the preparation of loaded NPs were decanted and their UV-Vis spectrum was obtained at 287 nm. The concentration of OLE was calculated using the calibration curve shown in Figure 2.

The percent EE was then calculated using Equation (2):

(2) $$EE\%=\frac{\mathrm{Tatal}\;\mathrm{amount}\;\mathrm{of}\;\mathrm{OLE}(\mathrm{mg})-\mathrm{Free}\;\mathrm{amount}\;\mathrm{of}\;\mathrm{OLE}\;\mathrm{in}\;\mathrm{superna}\;\tan\;\mathrm{ts}(\mathrm{mg})}{\mathrm{Total}\;\mathrm{initial}\;\mathrm{amount}\;\mathrm{of}\;\mathrm{OLE}(\mathrm{mg})}\times 100$$

#### 2.3.4. Thermal Properties

Differential scanning calorimetry (DSC) analysis was conducted on samples of OLE, PLA and OLE-loaded NPs using a Mettler DSC 1 STARe System^®^ (Mettler Toledo, Columbus, OH, USA). The specimens were heated from 30 to 170 °C, with a heating rate of 10 °C/min, under nitrogen flow (20 mL/min). The mass fraction crystallinity of the PLA (pure PLA, blank NPs) was calculated by Equation (3):

(3) $$x_{c}=\frac{\Delta H}{\Delta H_{0}}\times 100$$

where, ΔH is the heat of fusion of the sample (J/g) and ΔH_0_ is the heat of fusion of 100% crystalline polymer (J/g). ΔH_0_ is considered equal to 93.1 J/g [35].

#### 2.3.5. FT-IR Spectroscopy

FT-IR spectra of the OLE, the blank NPs and the OLE-NPs were obtained on a JASCO 4200 (JASCO Inc., Easton, MD, USA) using the ATR technique in the scanning range of 650–4000 cm^−1^.

#### 2.3.6. In Vitro Release Study

Preliminary in vitro release of OLE from the polymeric NPs was investigated using UV-vis spectroscopy, following a slightly modified literature procedure [38]. Experiments were carried out by suspending 12 mg of loaded nanoparticles into 4.2 mL of phosphate buffer (pH 5.6). The temperature was set at 37 ± 0.5 °C and the magnetic stirrer at 120 rpm. At appropriate intervals, the suspension was centrifuged at 12,000 rpm for 15 min. The supernatants were removed after every centrifugation and the precipitated nanoparticles were re-suspended in 4.2 mL of fresh buffer and were then put back in the magnetic stirring. The amount of OLE released in supernatants was inferred from the calibration curve used for the EE% calculation, in triplicate.

#### 2.3.7. Incorporation of OLE-NPs in Cosmetic Formulation and Stability Studies

The pure extract and OLE-loaded NPs were incorporated in an o/w base cream and stability tests were performed: for a 3-month period, samples were stored at different temperatures (5, 25, 40 °C and freeze-thaw cycles 5–45 °C) and were examined at regular intervals. The tested parameters of the stability studies were: viscosity, pH, organoleptic characteristics, emulsions phases and grid. The main characteristics of the o/w base cream used for this study were: light beige-yellow cream of characteristic scent, pH: 5.47, viscosity (25 °C): 20450cSt.

## 3. Results and Discussion

### 3.1. Antioxidant Activity of Olive Leaves Extract (OLE), PLA and OLE-NPs

Taking the multifactorial character of oxidative stress into account, we decided to evaluate the in vitro antioxidant activity of OLE, using two different antioxidant assays: (a) the radical scavenging ability of OLE was tested against the 1,1-diphenyl-2-picryl-hydrazyl (DPPH) stable free radical; and (b) the ability of OLE, OLE-NPs and PLA to scavenge hydrogen peroxide (H_2_O_2_), a very important reactive oxygen species the production of which is increased during skin aging, was tested using the luminol chemiluminescence method. Moreover, the total phenolic content of the OLE was determined. Quercetin, a potent antioxidant flavonoid which is used in many cosmetic formulations was used as the reference compound. The results are presented in Table 1.

The results show that the OLE prepared in this study is a potent DPPH radical and H_2_O_2_ scavenger, although less effective than quercetin, therefore it constitutes a promising additive for cosmetic applications. PLA does not show important antioxidant activity (IC_50_ 36.45 mg/mL), whereas the OLE-NPs present a significantly higher antioxidant activity than PLA (IC_50_ 4.37 mg/mL).

### 3.2. Phytochemical Profile of Olive Leaves Extract

**Figure 3:**
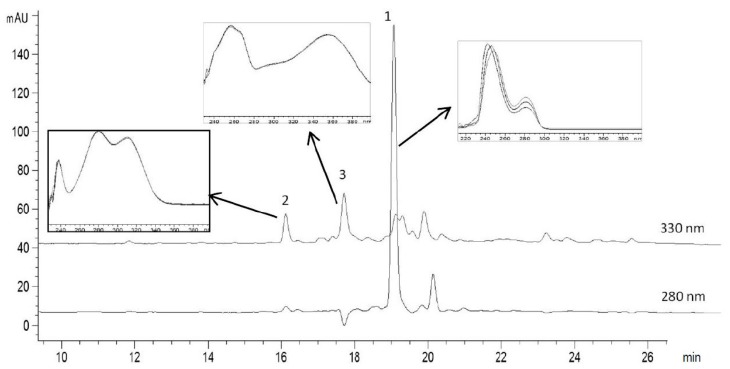
HPLC chromatogram of olive leaves extract (OLE). The peaks that were identified are: 1 oleuropein, 2 vanillin and 3 rutin. The UV-VIS spectra corresponding to the main polyphenols components are shown above the chromatogram.

The polyphenolic profile of olive leaves’ extract obtained from “Megaritiki” cultivar was determined using HPLC. A typical chromatogram of the olive leaves’ extract is given in Figure 3. Oleuropein, vanillin and rutin were identified by comparison of the UV-VIS spectra of the peaks separated by HPLC (Figure 3). Table 2 shows the retention times, the calibration equations with the corresponding coefficients of determination, the variation coefficients obtained in the consecutive analysis of 10 samples of each compound and the concentration of oleuropein, vanillin and rutin in OLE.

The quantitative determination of oleuropein, vanillin and rutin in OLE was achieved with a retention time of 18.4, 15.0 and 17.1 min, respectively. The sensitivity of the method was evaluated by determining the limits of detection (LODs) (signal-to-noise ratio (s/n) = 3) and the limits of quantification (LOQs) (s/n = 10). High coefficients of determination were obtained for all three standards (R^2^ = 0.9962, 1, 1 for oleuropein, vanillin and rutin, respectively) indicating good linearity response of the method proposed with LODs/LOQs (μg/mL) equal to: 44/135, 0.06/0.15 and 66/187 for oleuropein, vanillin and rutin, respectively.

The main phytochemical identified was the secoiridoid oleuropein (69.5%), as expected, because olive leaves are the richest source of this compound. Oleuropein content in OLEs can vary depending on plant maturation, cultivar type and harvest time. For example, Mourtzinos et al. found 90.2% oleuropein in the olive leaves extract obtained following exactly the same extraction procedure but another cultivar (“Kalamon”) harvested in the Thermopylae region (Central Greece) [15]. Small amounts of the flavonoid rutin and vanillin (4% and 1%, respectively) were also present in the studied extract. Rutin has been reported to be present in olive leaves extract obtained from the same Greek cultivar [39] whereas vanillin is a common phenolic compound in other Greek cultivars but, to our knowledge, has not been reported from the OLE extract from “Megaritiki” cultivar.

### 3.3. Nanoparticles (NPs) Characterization and Encapsulation Efficiency (EE%)

Particle size and polydispersity are the most important characteristics of nanocarrier systems. They determine the targeting ability of NPs and toxicity, while they greatly influence the drug loading, drug release and the stability of NPs [31,40]. In the present study, PLA NPs prepared in different batches showed mean particle size in the range of 166.8–291.2 nm and polydispersity indices between 0.08–0.26, revealing a homogenous nanoparticle population (Table 3).

**Figure 4:**
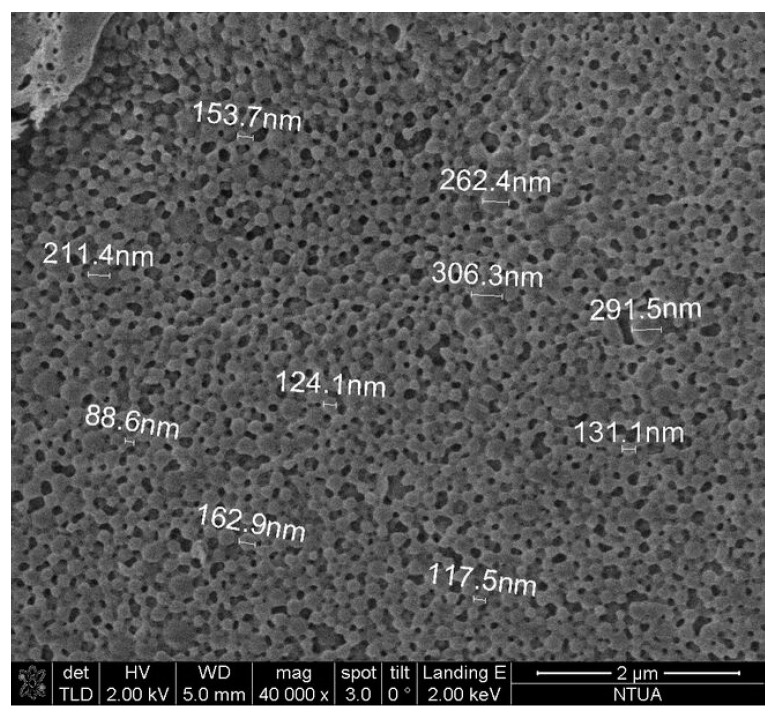
Scanning Electron Microscopy (SEM) image of PLA nanoparticles loaded with oil leaves extract (OLE-loaded NPs).

The morphology of discrete spherical polymeric nanoparticles was also verified by SEM indicating the effectiveness of the herein applied procedure to prepare OLE-loaded PLA nanospheres (Figure 4).

Zeta potential is also an important parameter in the characterization of NPs, since it measures the surface charge and gives information about suspension stability under defined conditions [24,31,41]. As can be observed in Table 3, the NPs prepared present negative ζ-potential values (−27.5 mV), which is indicative of a stable suspension without the tendency of aggregation. This value deviates from that of unloaded NPs (−19.3 mV), and this can be explained by the extract present on the surface of the NPs, as it is also shown in the FTIR-ATR spectra discussed below. Regarding the encapsulation efficiency of the extract, the loaded NPs showed a value of 49.2%. For comparison reasons, it can be cited at this point that higher EE value (82%) was reached by our group when using the same PLA grade as a carrier and a pure natural antioxidant (aureusidin) [24]. Obviously, the encapsulation of extracts, which are mixtures of compounds, presents a challenge regarding high EE values.

Νο quantification of residual methanol was performed in this study, as the main goal was to ensure the effective encapsulation of OLE extract to PLA nanoparticles, characterize the NPs and develop the encapsulation procedure. In the case that a commercial cosmetic product is going to be developed using these NPs, quantification of the residual methanol should definitely be performed in order to ensure that the product complies with international regulations.

### 3.4. Thermal Properties

**Figure 5:**
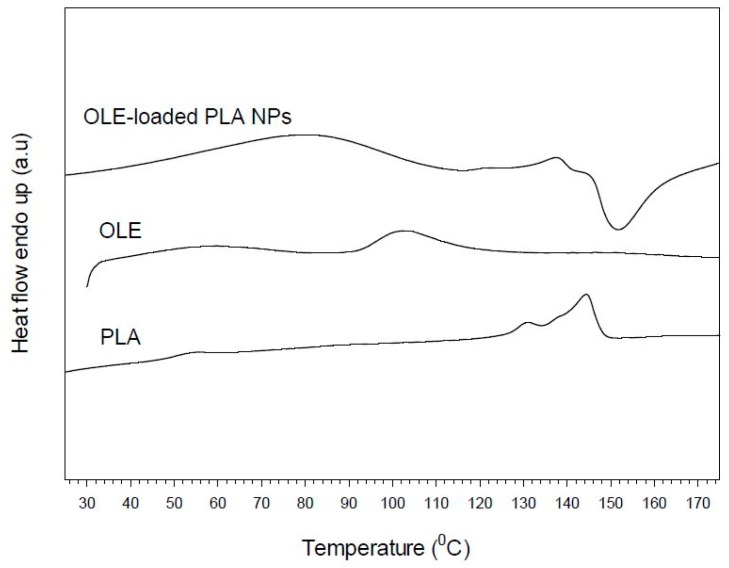
Differential scanning calorimetry (DSC) thermograms of pure poly(lactic acid) (PLA), olive leaves extract (OLE), and PLA nanoparticles loaded with oil leaves extract (OLE-loaded NPs).

DSC studies were performed in order to investigate the physical interactions between OLE and PLA in the formed nanoparticles. Different active substance/polymer combinations may coexist in the polymeric carriers, such as: (i) amorphous encapsulant in either an amorphous or a crystalline polymer and (ii) crystalline encapsulant in either an amorphous or a crystalline polymer [15,42,43,44]. Figure 5 shows the DSC thermograms of pure extract, pure PLA and loaded nanoparticles. The herein used PLA presented glass transition temperature (T_g_) at 51.4 °C and double melting behaviour with a small endotherm at 130.6 °C and a stronger one on at 144.3 °C. The mass fraction crystallinity was calculated at 34%. The extract (OLE) exhibited a broad endotherm in the range of 92–121 °C, which, however, disappeared in the DSC curve of the loaded particles. The latter may indicate the homogenous dispersion or dissolution of OLE in the polymeric matrix, as it was also found in the case of embelin-loaded polycaprolactone samples [45]. Similarly, the endotherm of PLA polymer became much smoother appearing at slightly lower temperature (138 °C), demonstrating the prevention of polymer crystallization during the nanoparticles formation and the prevalence of matrix amorphous state. Finally, in the case of loaded NPs, an exothermic peak appeared at ca. 150 °C following polymer melting, which may be attributed to morphological changes and/or degradation occurred to the encapsulated extract.

### 3.5. FT-IR Spectroscopy

**Figure 6:**
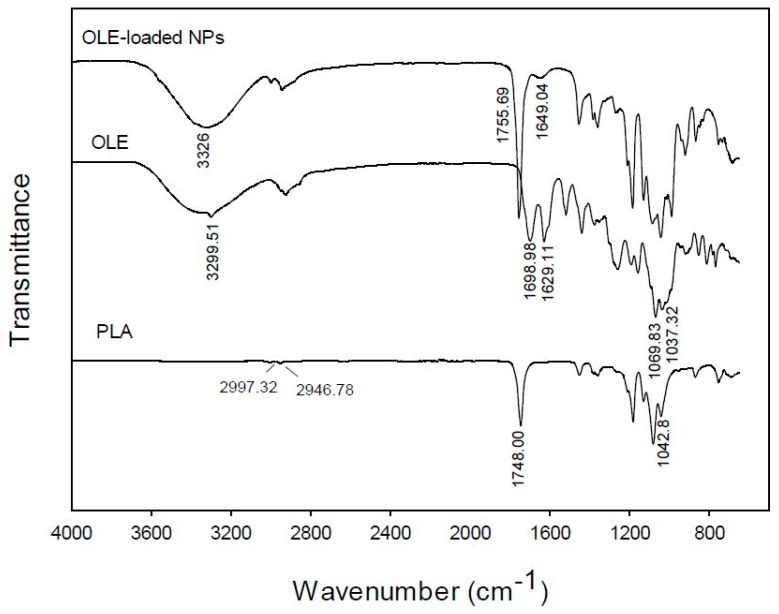
FT-IR (ATR) spectra of pure poly(lactic acid) (PLA), olive leaves extract (OLE), and PLA nanoparticles loaded with olive leaves extract (OLE-loaded NPs).

In order to obtain a better insight of the interaction between the encapsulated extract and PLA in the prepared NPs, the FT-IR (ATR) spectra of the free OLE, the polymer and the loaded NPs were obtained (Figure 6). The FT-IR spectrum of the pure extract is characterized by two absorption peaks at 1698.98 and 1629.11 cm^−1^, owed to the C=O stretching of the carbonyl groups of oleuropein, rutin and vanillin or other flavonoids which are present in the extract. The band at 3299.51 cm^−1^ is attributed to O-H stretching whereas the strong absorptions at 1069.83 cm^−1^ and 1037.32 cm^−1^ should be attributed to the C-O stretching of the ester groups present in the aforementioned phytochemicals of the extract.

In the FT-IR spectrum of PLA, the most characteristic peaks appear at 2997.99 and 2950.82 cm^−1^ owing to C-H stretching from the main chain of PLA, 1746.01 cm^−1^ characteristic of C=O stretching from the carbonyl groups of the repeated ester units and 1081.61 cm^−1^ attributed to the C-C(=O)-O stretching from the ester units.

The spectrum of the OLE-loaded NPs shows mainly the absorptions owed to the PLA polymeric matrix, which in most cases are overlapping with those of the encapsulated OLE [23]. However, two significant observations can be made: (a) in the spectrum of OLE-NPs there is a strong broad band at 3326 cm^−1^ and a weak absorption peak at 1649.04 cm^−1^, which can be attributed to the O-H stretching vibration of oleuropein as well as the other phytochemicals of the extract which are absorbed on the surface of the loaded NPs and are shifted in comparison to the spectrum of the pure extract (3299.51 and 1698.98, 1629.11 cm^−1^, respectively); and (b) the peak owed to the C=O stretching of the carbonyl groups of PLA is shifted from 1746.01 cm^−1^ in the spectrum of pure PLA to 1755.69 cm^−1^ in the spectrum of OLE-NPs. It can be postulated that the shift in the wavenumbers is owed to the interactions of the phytochemicals of the encapsulated extract with the PLA matrix.

### 3.6. In Vitro Release Study

**Figure 7:**
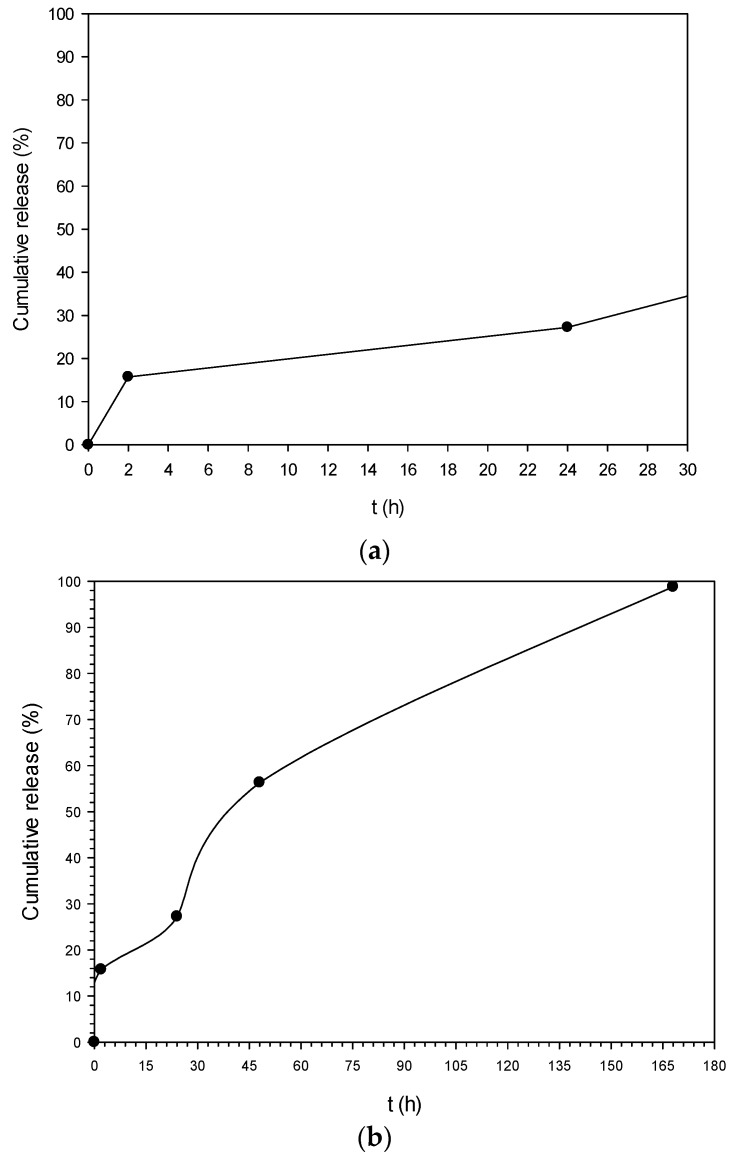
In vitro release profile of PLA nanoparticles loaded with oil leaves extract (OLE-loaded NPs) (pH 5.6, T = 37 °C). (**a**) 0–24 h; (**b**) 0–168 h.

Preliminary in vitro release experiments were conducted in pH 5.6 at 37 ± 0.5 °C. This pH value was selected as it is the pH of healthy skin [46]. The release profile is depicted in Figure 7a,b. At t = 2 h a burst effect was observed in which a cumulative amount of 15.7% of OLE was released (Figure 7a). After that, it is obvious that OLE “escaped” at a constant rate from the NPs reaching almost 100% cumulative release after 168 h (7 days) (Figure 7b).

### 3.7. Stability Studies

The results from the stability studies of the formulation incorporating the OLE-NPs prepared are shown in Table 4 and Table 5. There were no significant differences between samples referring to scent, rheology and emulsion phases. The samples with OLE showed changes in the colour and pH, indications of reactions that could lead to instability. The browning which occurred was most probably caused by the oxidation of the extracts’ polyphenols [10,19]. The results suggest that the formulation containing OLE-NPs is preferable since they do not affect the stability and appearance of the cosmetic emulsion.

## 4. Conclusions

The results of the current work demonstrated that the encapsulation of olive leaves extract in PLA NPs leads to nanoparticles-nanospheres with satisfactory characteristics, as initially observed in SEM images. The mean size of the particles formed was 246.3 ± 5.3 nm with satisfactory ζ-potential, polydispersity index and encapsulation efficiency. Moreover, based on the DSC and FT-IR studies, the olive leaves extract appeared to be protected inside the PLA matrix. The loaded NPs were successfully incorporated in a cosmetic emulsion without affecting its stability and appearance. Therefore, the encapsulation of sensitive polyphenolic extracts in biodegradable PLA nanoparticles provides the means to develop cosmetic formulations with advantageous characteristics.

## Figures and Tables

**Table 1:** Antioxidant activity of olive leaves extract (OLE), poly(lactic acid) (PLA) and OLE-loaded nanoparticles (OLE-NPs). Quercetin was used as a reference antioxidant.

	DPPH Radical Scavenging Ability IC_50_ (mg/mL)	Total Phenolic Content GAE (mg_gallic acid_/g_of dry extract_)	H_2_O_2_ Scavenging Ability IC_50_ (mg/mL)
OLE	0.283	391.7	0.254 ± 0.007
PLA	n.t. *	n.t.	36.45 ± 2.03
OLE-NPs	n.t.	n.t.	4.37 ± 0.12
Quercetin	0.073	n.t.	0.049 ± 0.003

* n.t. = not tested.

**Table 2:** High-performance liquid chromatography (HPLC) of olive leaves extract (OLE): validation parameters and quantification of the identified phenolic compounds.

Phenolic Compound	Retention Time (min)	Variation Coefficient (%) for Retention Time (n = 10)	Calibration Equation	Variation Coefficient (%) for Concentration (n = 10)	C (mg/mL)	% in OLE
Oleuropein	18.4	0.46	y = 5730.3x − 98.3 R^2^ = 0.9962	0.80	0.347 ± 0.035	69.5
Vanillin	15.0	0.19	y = 1755.5x + 3.51 R^2^ = 1	0.15	0.005 ± 0.001	1.06
Rutin	17.1	0.44	y = 824.9x − 1.53 R^2^ = 1	0.31	0.020 ± 0.005	4.03

**Table 3:** Characterization of indicative batches of blank PLA nanoparticles (blank-NPs) and PLA nanoparticles loaded with oil leaves extract (OLE-loaded NPs): particle size, polydispersity index (Pdi), ζ-potential, encapsulation efficiency (EE).

	Size (nm)	Pdi	ζ-Potential (mV)	EE%
OLE-loaded NPs	246.3 ± 5.3	0.21 ± 0.01	−27.5 ± 0.12	49.2
Blank-NPs	220.6 ± 4.0	0.08 ± 0.00	−19.3 ± 0.74	-

**Table 4:** Results of the pH measurements during the stability studies.

pH Results		Freeze Cycles				Storage at 5 °C	Storage at 25 °C	Storage at 40 °C		
Sample	Initial Results	Day 7	Day 15	Day 21	Day 29	Month 3	Month 3	Month 1	Month 2	Month 3
o/w Base Cream	5.47	5.49	5.5	5.52	5.48	5.6	5.49	5.47	5.37	5.48
Base Cream with OLE-NPs	5.49	5.59	5.54	5.56	5.52	5.62	5.59	5.48	5.35	5.43
Base Cream with OLE	5.45	5.5	5.5	5.46	5.42	5.55	5.5	5.41	5.3	5.26

**Table 5:** Results of the viscosity measurements during the stability studies.

Viscosity Results [cSt]		Freeze Cycles				Storage at 5 °C	Storage at 25 °C	Storage at 40 °C		
Sample	Initial Results	Day 7	Day 15	Day 21	Day 29	Month 3	Month 3	Month 1	Month 2	Month 3
o/w Base Cream	20,450	47,600	48,532	38,501	39,231	30,598	33,032	40,922	39,163	34,870
Base Cream with OLE-NPs	20,219	46,813	47,408	38,612	39,688	30,703	32,760	41,224	37,817	35,268
Base Cream with OLE	17,319	45,314	45,347	38,614	40,302	29,623	32,814	39,602	39,084	34,286
